# Abstracts and Selections

**Published:** 1909-05

**Authors:** 


### Abstracts and Selections.

### Carbolic Acid Gangrene.

Two observers writing from cities so remote from each other as St. Louis and Philadelphia have reported simultaneously five cases of gangrene following the use of carbolic acid as a surgical dressing. J. A. Kelley in the February number of *Annals of Surgery* reports a woman, aged 27, who applied a carbolic solution of unknown strength to a punctured wound of the finger. The dressing remained in place only five hours when intense pain brought about its removal. The finger was found to be dead and blackened from gangrene. The patient neglected advice of immediate amputation, but later consented to have the finger removed at the metatarso-phalangeal joint. In the *St. Louis Medical Review,* also for February, W. E. Leighton cites four cases of carbolic acid gangrene of fingers and toes. He considers that two factors are productive of this untoward result of the use of carbolic acid: the skin becomes hardened and contracted by the application and hence produces mechanical constriction after the manner of a very tight bandage; or an artery, already sclerotic, in the affected digit offers poor resistance against local necrosis.

The faith possessed by the laity in carbolic acid as a disinfectant and germicide leads them very frequently into error. In the first place there are many drugs equally or more powerful as antiseptics which do not have the local anesthetic effect or disagreeable odor of phenol. This anesthetic effect of phenol is one of its greatest dangers, for it is likely that when pain following its use does begin in adjacent tissue, irreparable damage has already been done. However, the layman with an injured member, upon visiting a nearby drug store, either demands a carbolic solution, or, as quite often happens, is supplied with the drug by the pharmacist who speaks only of its poisonous constitutional effect and neglects to warn the patient concerning its local action.

There is, then, sufficient reason for limiting the sale of this drug to physicians exclusively, and for not allowing it to be dispensed to almost anyone who is willing to sign the "poison book." Most surgeons of experience have seen cases of carbolic gangrene caused either by the patient's ignorance, or, in a few cases, by failure on the part of the physician who first saw the case to remember what may happen. Moreover, it is not the strong but the weak percentages of the drug that may do damage. A finger has been lost through the use of a 10 per cent carbolic ointment applied to an open wound, and a like result has followed the application of solutions 1 in 60 and 1 in 100 strength, respectively. In most hospitals, especially in the dispensary service, carbolic acid is used chiefly for the sterilization of instruments which are washed in 95 per cent alcohol and sterile water before use. Certainly in the light of past experience it is far safer to apply lead and opium, or alum acetate, or bichloride of mercury of suitable strength than to take chances with a drug so powerful in its local action as is phenol.—*Medical Record.*

---

### Wounds of the Skull.

Every suspicious wound of the scalp should be thoroughly explored to see that there is no fracture beneath; in case of doubt the wound should be enlarged for perfect inspection. The following rules laid down by Adams are to be carefully observed:

Gutter fractures should be invariably operated upon as early as possible to get rid of bone fragments, clots and debris, and to provide outlet for the products of infection which are common to these cases. Without early operation these wounds suppurate, and the operation is too late. (Von Manteuffel.)

In transverse perforating wounds clense orifices and await symptoms.

In superficial penetrating wounds with lodgments, exploration with removal of bone fragments and bullet should be made if practicable.

In transverse penetrating wounds cleanse the orifice of entrance, await symptoms, and employ radiography.

Cases of compression by blood-extravasation, with localizing symptoms, are rarely seen. When extravasation can be localized and is accessible, it should be treated on general surgical principles.

In some cases of penetration there may be fracture of the skull opposite the point of entrance, without exit opening.

Such a condition requires exposure and removal of the bone fragments; the bullet in such cases is rarely found near the second fracture, generally having been diverted to some other region by ricocet on the skull wall.

The question of removal of a bullet lodged in the cranium is one of special interest, inasmuch as a widespread belief prevails that a lodged bullet is *per se* a most dangerous thing. The danger lies in the damage done to the brain by the passage of the bullet through its substance, and, unless symptoms arise which can be traced to persistent irritation by the presence of the bullet, or radiography demonstrates that the bullet is in such position as to produce irritation sooner or later, no attempt should be made to remove a deeply seated bullet from the cranial cavity. Whenever we are confronted by the question of removal of a deeply lodged bullet, it should be definitely ascertained that the symptoms present are due to irritation from the presence of the bullet itself, and not to its having inflicted damage on certain regions of the brain on its way to its present location, for the removal of the bullet could in no way affect the injured cerebral tissue.—*Gaillard's Southern Medicine.*

---

THERE is less likelihood of injuring the deeper vessels in excising tonsils if the instrument is pressed in deeply to engage the organ rather than exerting pressure from the outside.—*American Journal of Surgery.*

---

### Tobacco and Sexual Power.—Cold Water to the Penis.—Heredity.

*Editor Medical World:*

Your reply on page 169 to an inquiry concerning a case of failing sexual powers is not as it should be—as I think, and I am butting in to remind you that a man who chews two ounces of tobacco per day is using enough of that drug to produce sexual paralysis, and the only way to help him as to this weakness is to wean him from the tobacco habit. I certainly find that a very common cause for this common complaint, and I think all your other good advice will fail to be satisfactory unless what I now say is accepted also.

Further, I would say that the application of very cold water for ten minutes at a time is too much of a good thing. A little of it is a rouser, but so much is depressing.

Then on page 163, I would certainly take exception to the writer who say that it makes no difference where the spermatozoa come from—"one sort is as good as another." Is there nothing in heredity?

New York, N. Y. E. B. FOOTE.

[For many years we have been accustomed to specify "five" or "ten" minutes for the cold water douche, with beneficial results. It is not the "rousing" effect that we wish, but the sedative action. We grant all you say regarding the effect of tobacco on *some men*, but we know many men who use two and three times this quantity daily, who are unimpaired. The effect of tobacco is not the same on all nervous systems, some tolerating a great deal more than others. It is always to be taken into consideration, when dealing with sexual debility, but we are somewhat cautious both in assigning the cause to the use of tobacco, and in forbidding its use, as we have noted results following the stopping of the habit which we felt were due to its abandonment. We are not advising anybody to contract the habit of using tobacco, but if it has been continuously used for twenty years, we do not think it should be lightly abandoned..—ED.]—*Medical World.*

---

DRYNESS of the pharyngeal wall is usually associated with an atrophic rhinitis.—*American Journal of Surgery.*

---

THE product, Chinosol, which is a definite chemical substance, being dioxychinolin sulphate, is relatively new to this country, though thoroughly well known in Europe. Its antiseptic strength is quite remarkable, even in spite of the fact that it is entirely non-poisonous. As an antiseptic it possesses many times the strength of carbolic acid and is fully equal to bichloride. It does not coagulate albumin, is non-caustic and non-irritating, does not damage the membranes and in addition is a local analgesic. It could not be presented in more convenient form. It comes in tablets which instantly dissolve in water. Any practitioner may carry the tablets with him, avoid the weight and nuisance of lugging a liquid with him and avoid the danger of poisoning some member of the household, in case by accident a tablet should be left in the sick room where a member of the household might get hold of it. It is now being introduced in America, and is supported by a wealth of clinical endorsement from eminent authorities in Germany, France, Austria, Italy and other European countries.

### Dirty Milk—The Remedy.

Few people are aware of the amount of dirt, and dirt of the filthiest kind, which is consumed in the use of ordinary milk. It was recently estimated that more than *twenty tons of cow manure are consumed as food disguised in milk by the inhabitants of the city of Berlin every year.* If this is true in Berlin, the amount consumed by an equal population in the United States must be much greater; for far less attention is given to sanitary supervision of such matters in this country than in Germany.

In a few States laws are now in force establishing a standard of purity for milk, or rather, we should say, a standard of impurity, for the standard is so low that milk which wholly conforms to it cannot be considered as in any wise clean. For example, in Michigan, where the standard is higher than in some States, the law demands that commercial milk shall not contain more than 200,000 microbes per cubic centimeter. A cubic centimeter is about one-fifth of a teaspoonful, so the actual meaning of the law is that a teaspoonful of commercial milk shall not contain more than one million germs, but of course it is impossible for inspectors to examine every specimen of milk offered for sale; consequently, *it is not an uncommon thing to find milk being distributed from door to door to be consumed by delicate invalids and feeble infants as well as by robust persons which contains as many as ten or twenty millions, or even fifty millions, of germs to the teaspoonful.*—From *Good Health.*

### Clinical Notes on Gonorrheal Cases Treated With Serum.

BY MELVIN S. ROSENTHAL, M. D.,
Associate Professor of Genito-Urinary Diseases and Dermatology, College of Physicians and Surgeons, Baltimore, Maryland.

While a large number of cases of gonorrhea are successfully treated in a comparatively short time without complications, it also happens that quite a large percentage of all cases are followed by complications such as prostatis, epididymitis, posterior urethritis and arthritis. All of these complications are stubborn and prone to successfully resist all forms of treatment. Arthritic cases especially are difficult to treat successfully and many of these patients become crippled or bed-ridden. Any new treatment which is based upon rational theories and appears to hold out hopes of successful treatment, merits consideration.

Rogers and Torrey call the attention of the profession to Antigonococcic Serum in articles appearing in the *Journal of the A. M. A.*, for January 27, 1906, and September 14, 1907. This serum is prepared from the blood of rams that have been treated with gradually increasing doses of both dead and live cultures of virulent strains of gonococci. The rams are treated until they are immune and the process of preparing the serum from their blood is essentially the same as that of preparing antidiphtheric and antitetanic serums. A quantity of this serum, which was placed in my hands for clinical experimentation, I have used in the treatment of 18 cases on male patients—four private patients and 14 in the Dispensary of the College of Physicians and Surgeons. The detailed histories of these cases would be of no particular interest, but as a contribution to the literature on the subject I offer the following resume of the results of the clinical study of this serum:

There were three cases of posterior urethritis which received a total of twelve injections with the result that one was promptly cured, one showed considerable improvement and one was unimproved.

Four cases of epididymitis received a total of ten injections; one was cured, two improved and one unimproved.

Four cases of prostatitis received a total of seven injections; two were decidely improved and two unimproved.

Seven cases of gonorrheal rheumatism received a total of twenty-two injections; two were cured, two were improved and three unimproved.

The dose given at each injection was the contents of one bulb of 2 ccs. of serum. This was repeated at intervals of one to four days, controlled by the clinical symptoms. The injection was made deep into the muscular tissues.

A recapitulation of the eighteen cases treated is as follows:

Four (or 22½ per cent.) were cured.

Seven (or 38½ per cent.) were improved.

Seven (or 38½ per cent.) were unimproved.

—*Journal-Record of Medicine, Atlanta, Ga.*

---

Hard tonsils preponderating in connective tissue, are better removed by the cold snare than by a sharp instrument. The snare closes the blood nerves; the tonsillitome open them.—*American Journal of Surgery.*

AN HYPERTROPHIED lingual tonsil sometimes causes much discomfort, giving a heavy, sore feeling to the base of the tongue. It may be necessary to remove it.—*American Journal of Surgery.*

### Appendicitis in General Practice.

This paper deals with the subject not for the specialist, but for the general practitioner. There is no disease that makes greater demand on the vigilance and presence of mind of the practitioner. In their analysis of thirty-four cases they mention the fact that the majority have occurred in very healthy individuals as well as twenty of the thirty-four in women. Most of the cases were in young patients and one gave the history of a blow on the right flank.

One point of interest is that they advise whether the pus is localized or free in the peritoneal cavity, the preparation of a vaccine to hasten the healing.

Among the symptoms one rule is that every case with abdominal symptoms, to which no definite cause can be assigned, should be regarded as suspicious. As a rule the patient complains of pain, which may be localized over McBurney's point or somewhere near, may be severe in the pit of the stomach, or high up on the right side of the abdomen or general over the abdomen; it may be in spams or as a constant aching about the appendix, these two being most common. The maximum tenderness may be over the stomach or right kidney, and flatulence may be present. There may or may not be nausea and vomiting. Tongue is dirty and a history of constipation, but may be diarrhea. In women, be certain about menstruation, because any tenderness will be increased in this case. Fever may be absent at first, or they may run an apyrexial course; pulse rate increased. The symptoms may be due to gastro-intestinal disturbance, arising from a chill, or from indiscretion in diet. In some cases tenderness may be felt per rectum when not revealed externally.

It is sometimes impossible to diagnose perforation for several hours, but the practitioner has a better opportunity of making this diagnosis, if he is seeing the patient several times a day, than the surgeon at one examination.

Perforation may declare itself by means of pain so violent as to almost produce fainting, and may be followed by a rigor and sudden rise of temperature, or it may be uhsered in by a lull in the symptoms and a cessation of pain and discomfort, so much as to make one think the patient is better.

If the temperature does not rise, and the pulse gradually increases, the prognosis is grave; before death there is often an antemortem rise of temperature; the wiry pulse is seen late.

There is no medical treatment for appendicitis, and every case should be regarded from the surgical point. The diet should be limited to sips of hot water and hot applications locally. Morphia may be given, if it has been decided to call a surgeon, but if the patient refuses to see a surgeon at once, then it is better avoided. It is the duty of the general practitioner to bring the surgeon to the case early.—*Southern Medical Journal.*

---

SUPPURATING ARTHRITIDES does not always require exposure of the joint or even large incisions, irrigation and drainage. Such treatment invites mixed infection and ankylosis. If the pus be very thin—even though of streptococcic origin—thorough *aspiration* (which may need to be repeated) and immobilization may effect a rapid cure with perfect function. Purulent arthritis and periarthritis as it occurs in small children as a complication of one of the exanthemata (often in connection with trauma) is often quite amenable to conservative, and even ambulant treatment: aspiration, or irrigation and drainage, and immobilization. Judgment is needed, of course, to determine what cases are amenable to this conservative surgery, and what point in the treatment it must be abandoned in favor of more extensive intervention.—*American Journal of Surgery.*

---

### A New Heat Stroke Theory.

Under the subject of Military Medicine (*Deutsche Militärärztliche Zeitschrift*) Dr. Hiller discusses Dr. Senftleben's recent theory of heat stroke, which may be briefly stated as follows: In consequence of the continual perspiration of the soldiers on the march, the red blood corpuscles undergo dissolution. The hemoglobin of the red blood cells, dissolved in the blood plasma, destroys the white blood corpuscles, and liberates, from the protoplasm of the white cells, the fibrin ferment, which calls forth an active rise in temperature and coagulation of the blood, especially in the venous system, and by obstructing the pulmonary artery and its branches leads to death with lightning rapidity. The severity of the attacks of heat stroke depends upon the amount of fibrin ferment produced and the varied intensity of coagulation. The experimental support of Senftleben's theory are the experiments of Maas; "Influence of the rapid withdrawal of water from the organism," and three dissertations of Koehler, Sachsendahl, and Maissurianz; "Fibrin ferment and hemoglobin intoxication," from Schmidt's laboratory in Dorpat. Hiller made a thorough study of the investigations of Maas and the dissertations from Schmidt's laboratory and comes to a widely different conclusion. He can not see how the perspiring of soldiers on the march can be compared to the withdrawal of water caused by exposing in a room of warm, dry air the contents of the abdominal cavity of a narcotized rabbit, nor do the injections of a concentrated sugar solution into the peritoneal cavity of a rabbit or dog confirm Senftleben's theory to him. The experiments of Koehler, Sachsendahl, and Maissurianz were made to explain blood transfusion. They found that dissolved hemoglobin, prepared from the blood of an animal, when injected in the jugular vein of the same or any other animal, led to a rapid disintegration of the colorless blood cells and a sudden accumulation of a large amount of fibrin ferment, which caused coagulation of the venous blood in the lesser circulation and produced sudden death by asphyxiation. Since Senftleben's theory requires coagulation of the venous blood in the lesser circulation, then one should find clots in the pulmonary artery and its branches in a patient dead from heat stroke. No such findings have as yet been reported. Sentfleben's explanation that the fibrin ferment has been used up does not explain the absence of a coagulation.—*The Military Surgeon.*

---

THE Twelfth International Anti-Alcoholic Congress will be held in London from July 18th to the 24th. Field Marshal the Duke of Connaught is the honorary president. The work of the congress will include several inaugural addresses by distinguished persons from this country and Europe, and many sectional meetings will be held in which the special phases of the subject will be discussed. Representatives from all the civilized countries will be present, and an exhibition of publications and matters pertaining to the alcoholic question will constitute a very prominent feature of the occasion. In all probability this will be the largest congress ever held, and delegates from this country representing every society and organization interested in the alcoholic problem will be welcomed.

Programs and information can be had at the offices of the National Temperance League, 34 Paternoster Row, London, E. C., England.

There will be ample opportunity for the reading of papers by any credited representative of this country, the particulars of which will appear in circulars later.—*Journal Inebriety.*

---

The best point for entering the maxillary antrum is about one inch from the edge of the nostril, below the inferior turbinate.—*American Journal of Surgery.*

---

### The Use of Antiseptics in Gynecology, With Special Reference to Utero-Vaginal Catarrh.

BY M. LUTHER SPRIGGS, M. D., JOPLIN, MO.

During recent years much has been said and written concerning the value of antiseptics and the wonderful changes wrought by their use have been little short of marvelous.

A large percentage of the pelvic diseases occurring in the female are of bacterial origin and the recognition of this fact and the application of the modern principles of alkaline antiseptics are responsible for the relief of many cases which were formerly a source of great anxiety to both patient and physician because of their intractability.

Another important field of usefulness for alkaline antiseptics which should not be overlooked in gynecologic practice and which is second only in importance to the relief of already existing pathologic conditions, is in the prevention of disease, or when such disease already exists in limiting its action to the minimum. As an example many cases of utero-vaginal catarrh simple enough in themselves will, if neglected or improperly treated, terminate in complications of a very serious nature.

There is not the slightest doubt but that many of the inflammatory diseases to which women are liable may be prevented by absolute cleanliness and the use more or less regularly of a suitable antiseptic.

We have at our disposal numerous agents possessing decided antiseptic properties, but unfortunately the usefulness of many of these is limited by other properties of an objectionable nature.

The use of any remedy which has a tendency to produce induration or thickening of the tissues with which it comes in contact, should be discouraged, as should also those agents which, while decidedly antiparasitic in their action, are too irritating to be used with safety on the delicate tissues of the vagina.

In this connection the writer desires to call attention to Glyco-Thymoline, which fulfills all the requirements for an active and reliable antiseptic of proper alkalinity, while at the same time it is free from the objectionble features so common to remedies belonging to this class.—*Southern Practitioner.*

